# Effects of TMEM232 Variant on Infant Atopic Dermatitis According to Maternal Factors

**DOI:** 10.3390/genes15111446

**Published:** 2024-11-08

**Authors:** Eun-A Choi, Hee-Soo Han, Guemkyung Nah, So-Yeon Lee, Young Youl Kim, Soo-Jong Hong, Hye-Ja Lee

**Affiliations:** 1 Division of Allergy and Respiratory Disease Research, Department of Chronic Disease Convergence Research, Korea National Institute of Health, Korea Disease Control and Prevention Agency, Cheongju 28159, Republic of Korea; euna9508@korea.kr (E.-A.C.); heesu3620@hanmail.net (H.-S.H.); forest@korea.kr (G.N.);; 2 Department of Pediatrics, Childhood Asthma Atopy Center, Humidifier Disinfectant Health Center, Asan Medical Center, University of Ulsan College of Medicine, Seoul 05505, Republic of Korea; imipenem@hanmail.net (S.-Y.L.); sjhong@amc.seoul.kr (S.-J.H.)

**Keywords:** TMEM232, atopic dermatitis, maternal factor, total IgE

## Abstract

**Background:** Atopic dermatitis (AD) is caused by interactions between genetic susceptibility and environmental factors. Transmembrane protein 232 (*TMEM232*) is one of the genes strongly implicated in AD. **Methods:** In the present study, we aimed to investigate the association between AD with variants within *TMEM232* based on maternal factors, including a history of allergic diseases, and sensitization to Der f. We performed a candidate gene association study involving the Cohort for Childhood Origins of Asthma and Allergic Diseases. **Results:** A single variant of the *TMEM232* gene, rs17132261, was found to be significantly associated with AD. Subjects carrying the wild-type allele (C) of rs17132261 had higher total IgE than those carrying the variant rs17132261 (T). Multiple logistic regression analysis showed a statistically significant association between *TMEM232* gene polymorphism and an increased risk of AD in one-year-old infants. Moreover, rs17132261 was associated with increased total IgE in infants with a maternal history of allergic disease. The group with the CC genotype showed a higher risk of developing AD compared to carriers of CT and TT genotypes when the mother had a history of allergic diseases or was sensitized to Der f. **Conclusions:** Our findings demonstrate that the *TMEM232* risk allele, in combination with maternal factors, higher the total IgE, which could be a potential risk factor for AD.

## 1. Introduction

Atopic dermatitis (AD) is a multifactorial chronic disease characterized by skin inflammation caused by both genetic and environmental factors [1,2]. The prevalence of AD is highest among children, with the prevalence of AD in infants ranging from 10% up to 20% in Korea [3,4]. Global Burden of Disease data confirm that AD affects patients’ quality of life and has a high global burden [5].

Maternal history of allergic diseases and exposure to house dust mites (HDMs) during pregnancy are recognized as significant risk factors for the development of AD in offspring. Several studies show that maternal history of allergic diseases has been strongly linked to the development of AD in infants, with immunoglobulin E (IgE) playing a key role in this process [6,7,8]. Prenatal exposure to HDM allergens has been linked to an increased risk of AD in offspring, suggesting that maternal HDM exposure during pregnancy may enhance allergic susceptibility through vertical transmission of immune responses. A study demonstrated that prenatal exposure to HDM allergens influences T cell phenotypes and cytokine production, contributing to AD development in the first year of life [9]. Additionally, a study indicated that maternal HDM exposure increased the offspring’s susceptibility to allergies in murine models, underscoring the importance of maternal immunity in shaping offspring immune system [10].

Genome-wide association studies (GWAS) have identified 62 genes associated with AD, most of which encode skin barrier proteins involved in the innate and adaptive immune responses [11]. *TMEM232* (transmembrane protein 232), located on chromosome 5q22.1, is strongly associated with AD [12,13,14]. Recently, a haplotype analysis revealed that the H15 haplotype in the 5q22.1 region was associated with functional mutations in the *TMEM232* gene [15]. *TMEM232* has also been implicated in the development of several allergic diseases, the underlying mechanisms of which are thought to involve blood components. Additionally, some Asian populations, including Chinese and Japanese populations, are especially susceptible to allergic diseases owing to variations in *TMEM232* [12,13]. However, an association between cord blood and the susceptibility to allergic diseases has not yet been reported in the Korean population. Here, we investigated the association between the *TMEM232* variant and blood parameters related to AD using Korean birth data and examined the potential relevance of *TMEM232* variants in early childhood AD.

## 2. Methods

### 2.1. Study Participants

**Figure 1:**
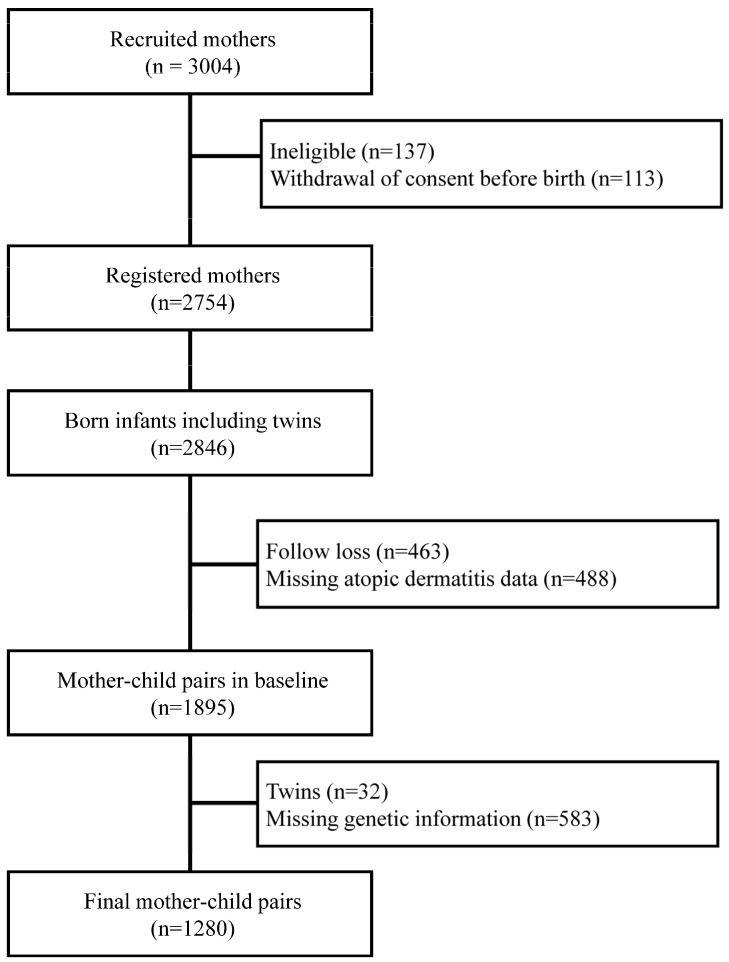
Participant inclusion and exclusion criteria.

This study analyzed the Cohort for Childhood Origin of Asthma and Allergic Disease (COCOA), a prospective longitudinal study that aimed to investigate the effects of environmental and genetic factors on the development of allergic diseases. The COCOA study recruited 3004 mothers from the general population before 26 weeks of gestation [16]. Of these, 2846 children were born, and regular follow-up visits for health and environmental questionnaires and physician examinations were conducted at 6 months, 1 year, and yearly thereafter. The participants who were ineligible (*n* = 137), withdrew consent (*n* = 113), were lost to follow-up (*n* = 463), or had missing AD information (*n* = 488) were excluded, resulting in a total of 1895 mother–child pairs. Baseline characteristics were then assessed using these 1895 pairs. After further excluding individuals without genetic information, 1280 mother–child pairs remained for subsequent analysis (Figure 1). The number of participants included in the analysis for each clinical variable varied due to the presence of missing values. We have presented the number of participants for each variable in the tables.

### 2.2. Questionnaire Data

The questionnaire, which was based on the International Study of Asthma and Allergies in Childhood (ISAAC) [17], was translated into *Korean*. The parents of the infants filled in a modified version of the questionnaire. The questionnaire consisted of (1) demographic factors, such as sex, date of birth, height, and weight; (2) symptom history related to AD; and (3) environmental factors related to allergic diseases, such as feeding method and residential patterns [18].

Familial history of AD was defined using a questionnaire conducted when the mother was 26 weeks pregnant.

### 2.3. Skin Prick Test

A skin prick test (SPT) was performed in the mother within 6 months after child’s birth. The SPT was conducted for 14 allergens (mites, molds, pollens, animal dander, and cockroach). Normal saline and histamine dihydrochloride were used as a negative and positive control, respectively. A mean wheal size greater than 3 mm was considered a positive reaction.

### 2.4. Definition of Atopic Dermatitis

AD in infants was evaluated through questionnaires at each scheduled visit, inquiring whether a physician had ever diagnosed AD and if so, whether interventions were prescribed for the condition. AD was defined according to criteria described by Hanifin and Rajka. The AD phenotype of the infants was considered at one year of age. A pediatric allergist assessed the SCORing Atopic Dermatitis (SCORAD) index [19]. Higher numbers indicate greater severity, and the scale ranges from 0 to 103.

### 2.5. Measurement of Leukocytes and IgE

White blood cell counts and IgE levels (total IgE and milk-specific IgE) were measured. The percentage of blood leukocytes was calculated by using an automatic blood cell counter (XE-100; Sysmex Co., Kobe, Japan). Total serum IgE and milk-specific IgE levels were measured using a fluorescent enzyme immunoassay (AutoCAP System; Pharmacia Diagnostics AB, Uppsala, Sweden). Leukocyte and IgE levels in blood from infants at one-year-old were measured.

### 2.6. Genotyping

Blood samples were collected from each participant, and the derived cord blood DNA samples were genotyped using the Korea Biobank Array (KoreanChip) designed by the Korean National Institute of Health, Korea. The KoreanChip comprises 833,535 markers, including more than 247,000 rare frequencies or functional variants derived from the sequencing data of 2500 *Koreans*. Genotyping data were removed for low-quality SNPs with a low variant call rate (< 98%), excessive heterozygosity, and sex discrepancies [20].

The association between AD and *TMEM232* has been well established in previous studies [12,13,14]. We examined the association of 180 SNPs located in *TMEM232* with early-onset AD, identifying 10 SNPs with significant associations (Supplementary Table S1). Among these, we selected the SNP rs17132261, which demonstrated a strong association, for further analysis. The minor allele(T) frequency of the SNP rs17132261 is 0.269.

### 2.7. Statistical Analysis

All data are expressed as mean ± standard deviation (SD) for continuous variables and as frequencies and proportions for categorical variables. To compare groups, *t*-tests and ANOVA were conducted for continuous variables, and the chi-square test was performed for categorical variables. To investigate whether *TMEM232* genetic variants were related to AD, odds ratio (OR) and 95% confidence intervals were calculated based on logistic regression. We performed linear regression analysis to determine whether blood leukocytes and IgE are associated with the genotype of *TMEM232* rs17132261. Additionally, we conducted further analysis stratifying for the environmental factors associated with AD. The adjusted variables used were maternal age, and feeding method. All *p*-values were two-tailed, and *p*-values below 0.05 were considered statistically significant. All statistical analyses were performed using R software (version 4.1.3, R Project for Statistical Computing, Vienna, Austria).

## 3. Results

### 3.1. Characteristics of the Study Participants

Participants’ characteristics are presented in Table 1. A total of 496 infants were diagnosed with AD by doctors. The mean eosinophils were 2.94 in the non-AD and 3.39 in the AD group; that is, the values were significantly different. The mean concentrations of total IgE and specific IgE to egg were 97.26 and 3.55 in the AD group, respectively, while they were 56.11 and 0.81 in the non-AD group, respectively. Both total IgE and specific IgE to egg showed significant differences when comparing the AD group to the non-AD group. The maternal eosinophils were higher in infants with AD and showed significant differences according to the AD status of their offspring. The sensitization rate to Der f among mothers was 43.0% in the AD group compared to 38.2% in the non-AD group, suggesting a notable trend of difference between the two groups. Similarly, the history of allergic diseases in mothers was 34.9% in the AD group versus 30.5% in the non-AD group, indicating a tendency for differences. In addition to the sensitization to house dust mites and history of allergic disease, maternal prenatal environmental factors such as pets, alcohol consumption, secondhand smoke exposure, and mode of delivery were also considered; however, none of these factors showed an association with the child’s AD (Supplementary Table S2).

### 3.2. Association Between TMEM232 Genotype and AD

We analyzed the association between the genotype of *TMEM232* variant rs173132261 and AD using logistic regression (Table 2). We found that infants with the TC (n = 498) and CC (n = 687) genotype had a higher adjusted odds ratio (aOR) for the incidence of AD than infants with the TT (n = 95) genotype (aOR 2.77; 95% CI 1.39–6.51; *p*-value = 0.004, aOR 3.45; 95% CI 1.75–6.79; *p*-value < 0.001). The incidence of AD was higher in individuals with the C allele than in those with the T allele.

### 3.3. Parameters for the Study Participants According to TMEM232 Genotype

Table 3 lists the descriptive statistics for the study participants included in the analysis based on their *TMEM232* genotype. We investigated whether the AD-related blood biomarkers were associated with *TMEM232*. As shown in Table 3, there were significant differences between the infant’s lymphocytes percentages in the three groups. Additionally, after adjusting for maternal age and feeding method, maternal Der f IgE demonstrated significant differences among the groups, with higher values observed in those carrying the C allele. The infant’s total IgE also showed a slight difference, with values of 64.02 for the CC genotype, 66.18 for the TC genotype, and 47.33 for the TT genotype. This indicates that the TT genotype, associated with a lower risk for AD, had the lowest total IgE levels.

### 3.4. Association Between Biochemistry Parameters and Maternal Factors

We summarized the descriptive statistics of biomarkers according to maternal factors associated with AD, such as history of allergic diseases and sensitization to Der f. In cases where mothers had a history of allergic diseases, infant’s specific IgE to egg levels were observed to be higher. Additionally, in instances of maternal sensitization to Der f, the infant’s monocyte levels were higher, while their lymphocyte levels were lower, showing differences between the groups.

### 3.5. Impact of TMEM232 and Maternal Factors on Offspring’s Clinical Data

We compared the children’s blood biomarkers considering the *TMEM232* genotype alongside maternal history of allergic diseases and sensitization to Der f (Table 4). The children’s total IgE levels were 76.81 and 59.23 for those with the C allele, depending on their mothers’ history of allergic diseases, while levels for those with the TT genotype they were 52.34 and 43.99, respectively, based on maternal allergic disease history. In children with the C allele, monocyte levels were measured at 8.28 and 7.94 based on their mothers’ sensitization to Der f. In contrast, those with the TT genotype had monocyte levels of 8.22 and 7.26, respectively, depending on maternal sensitization status. We observed significant differences in total IgE and monocyte levels after adjusting for maternal age and feeding method in relation to children’s AD risk factors, including the *TMEM232* C allele, maternal history of allergic diseases, and sensitization to Der f.

### 3.6. Association Between TMEM232 Genotype and AD According to Maternal Factors

Classified by maternal environmental factors, the association between *TMEM232* genotype and the risk of AD in infants was investigated (Table 5). When analyzing the association based on the maternal history of allergic diseases, the highest aOR was observed in individuals without such a history for the CC genotype at 2.70. Among those with an allergic disease, the risk increased, showing an aOR of 5.78 for the TC genotype and an even higher level of 6.69 for the CC genotype. Additionally, when the association was analyzed by dividing the maternal Der f-IgE levels by a sensitization cutoff of 0.35 IU/mL, it was determined that, in children of mothers who were not sensitized to Der f, the aOR for the CC genotype was 3.72. Among children of sensitized mothers, the aOR increased to 4.85 for the TC genotype and reached 4.91 for the CC genotype. Therefore, the risk of AD in infants increases when mothers have a history of allergic diseases or are sensitized to Der f, particularly with a presence of *TMEM232* rs17132261 C alleles.

## 4. Discussion

We found that the association between the *TMEM232* variant and maternal factors has an effect on the development of AD in this cohort. Infants carrying the wild-type allele C of *TMEM232* rs17132261 showed an increased risk of AD and higher total IgE levels due to maternal factors such as susceptibility to allergic diseases compared with those carrying the T allele.

A relationship between maternal immune status and allergen sensitization to offspring’s allergic responses has been proposed previously [21,22,23]. One study identified an association between the maternal and child levels of cytokines that regulate IgE and eosinophils [24,25]. The maternal levels of inflammatory cytokines such as interleukin-10 and TNF-α during pregnancy have been reported to be correlated with the levels of the corresponding cytokines in children at 1 year of age [25]. In addition, maternal total IgE and allergen-specific IgE levels were predictive for cord blood IgE as well as IgE in infants at 1 year of age [25]. The maternal immune system is also linked to atopy in offspring during their first year of life [23,25]. As such, studies have confirmed the relationship between maternal family history of atopy including AD and total IgE in and infant AD; our birth cohort study showed that the higher the number of eosinophils in the mother, the higher risk of AD in the young child. Here, we suggest that inflammatory indicators, like lymphocytes, eosinophils, and Der f-specific IgE, show a correlation between mothers and children early in life.

AD pathogenesis is assisted by an ensemble of both genetic alterations related to the epidermal barrier and immune system and gene–environment interactions. The detection of AD in early life and the identification of genetic factors that interact with innocuous environmental factors are important for a timely diagnosis. In the present study, we identified *TMEM232* as a candidate gene for AD in Korean infants by analyzing single-nucleotide polymorphisms reported in previous genome-wide studies on AD. Furthermore, we studied the association of *TMEM232* genotypes and AD-associated phenotypes with increased IgE levels. We observed an association between the *TMEM232* variant rs17132261 (C > T) in the cord blood and Der f. IgE in the mother. The results indicate that the C allele of rs17132261 is a risk factor for infant AD when the mother has a history of allergic diseases and higher level of Der f.-specific IgE. It may manifest its effects through an increased total IgE alongside a maternal history of allergic disease and increased monocytes in maternal sensitization to Der f.

Previous studies suggested that *TMEM232* is a potential genetic risk factor for AD. A GWAS conducted at the UK Biobank Europeans confirmed the association of *TMEM232* with allergic diseases [26], immunoglobulin type E in grass [27], and AD [28,29]. Moreover, Japanese and Chinese Han GWASs have reported *TMEM232* as a novel gene associated with AD [12,13]. Additionally, locus 5q22.1, which includes the AD-associated gene TMEM232, has been reverified [28] and thoroughly sequenced in the Chinese population [15]. Genetic variants of *TMEM232* are associated with several allergic diseases, including AD [27,30,31,32]. A study on European participants confirmed an association between *TMEM232* and allergic rhinitis [27]. *TMEM232* has been confirmed to be associated with childhood food allergies [30] and asthma [31]. In this study, we confirmed the association between infant AD and the *TMEM232* variant rs17132261, which is mediated by a maternal history of allergic disease and sensitization to Der f.

Transmembrane proteins (TMEMs) are firmly attached to cellular membranes. TMEMs play crucial roles in cellular physiology, including transportation, signal transduction, and intercellular communication [33,34,35]. Although the specific functions of TMEM family proteins are still poorly characterized, new findings have highlighted the importance of the TMEM family as prognostic markers for various diseases [36,37,38]. Among TMEM family proteins, *TMEM45A* has been reported to be correlated with epidermal keratinization through the Golgi network in both in vivo and in vitro models [39]. *TMEM79* is a predisposing gene for AD which is related to the lamellar granule system, mast cell degranulation, and skin barrier function [40,41,42]. *TMEM232* has also been identified as a susceptible gene for AD according to several GWASs [12,13,15,29], the molecular function of which has recently been investigated [41]. Exposure to Der p activates signaling pathways involving PI3K, AKT, and NF-κB, leading to the downregulation of filaggrin expression and the upregulation of pro-inflammatory cytokines [43]. These cytokines promote T cell maturation, a process in which the RAG2 gene plays a crucial role [44]. *RAG2* and *TMEM232* were identified as putative related factors, which were found to interact in other organisms [45]. Following maturation, T cells differentiate into activated Th2 cells, further contributing to the inflammatory milieu characteristic of atopic dermatitis. Recently, Han et al. reported that *TMEM232* is overexpressed in the skin tissues of AD patients and mice, promoting inflammatory responses via the NF-κB and STAT3 pathways, which exacerbates the inflammatory environment and results in the formation of AD-like lesions [46]. Wu et al. reported the expression of *TMEM232* in the granular layer and weak expression in the basal layer of skin samples from Chinese patients with AD using immunohistochemical staining [29]. Collectively, these studies suggested that *TMEM232*, a gene that regulates AD-related inflammation, could be a target for AD treatment

*TMEM232* was confirmed to be related to AD as a genetic factor, and a correlation showed that maternal history of allergic disease and sensitization to Der f. increased the risk of AD as an environmental factor. Several studies consistently reported that a maternal history of allergic disease, such as asthma or atopic dermatitis, contributes to an increased risk of developing AD. A study has demonstrated shared prenatal impacts among childhood asthma, allergic rhinitis, and AD [47], supporting the role of maternal influences in early immune system development. A systematic review further confirms that a parental history of atopic disease is a strong predictor of AD in offspring [48]. Additionally, molecular networks in atopic mothers have been found to influence the infant’s risk of developing AD, illustrating the intricate relationship between maternal factors and the susceptibility of offspring to allergies [49].

The present study is valuable because it is easy to translate the obtained results to the general population because we analyzed the disease phenotypes of infants in a natural birth cohort and not in a case–control study. In particular, this is the first study to confirm the association between infant *TMEM232* variants, maternal factors, and offspring’s total IgE and AD in the Korean population.

## 5. Conclusions

In conclusion, this study reports that the *TMEM232* gene in cord blood is associated with infant total IgE, which is important in normal immune responses and preventing allergic diseases. Moreover, the *TMEM232* variant rs17132261 may be implicated in the maternal history of allergic disease and sensitization to the Der f-mediated pathogenesis of AD and other allergic diseases. Determining the association between *TMEM232* genotype and AD may provide insights into the biological mechanisms underlying AD.

## Figures and Tables

**Table 1:** Characteristics of mothers and one-year-old infants with atopic dermatitis.

	Non-AD		AD		
	N	Mean ± SD or %	N	Mean ± SD or %	*p*-Value
Infant					
WBC	1125	8.97(±2.59)	365	9.51(±3.00)	0.001
Monocytes, %	1119	7.95(±2.91)	365	7.79(±2.83)	0.367
Lymphocytes, %	1119	62.77(±10.65)	365	62.07(±11.23)	0.284
Neutrophils, %	1118	25.74(±10.30)	363	26.02(±10.48)	0.651
Eosinophils, %	1118	2.94(±1.84)	365	3.39(±2.88)	0.001
Basophils, %	1116	0.52(±0.34)	364	0.54(±0.45)	0.426
Total IgE	1116	56.11(±137.76)	355	97.26(±266.59)	<0.001
Egg IgE	1116	0.81(±2.83)	355	3.55(±11.56)	<0.001
Feeding method	1236	92.7	442	91.5	0.447
Mother					
Age	1394	33.23(±3.57)	490	33.42(±3.56)	0.298
WBC	1126	8.89(±2.89)	380	8.80(±2.69)	0.615
Monocytes, %	882	6.82(±1.90)	295	6.74(±2.08)	0.522
Lymphocytes, %	885	21.43(±8.67)	295	20.44(±7.91)	0.084
Neutrophils, %	873	70.15(±10.23)	287	70.78(±8.92)	0.349
Eosinophils, %	880	1.13(±1.07)	288	1.33(±2.13)	0.034
Basophils, %	878	0.21(±0.16)	287	0.21(±0.17)	0.836
Total IgE	1084	106.18(±267.45)	380	118.98(±291.51)	0.433
Der f-IgE	915	4.55(±11.31)	308	5.31(±13.19)	0.326
SPT Der f	478	38.2	178	43.0	0.097
History of allergic disease	427	30.5	173	34.9	0.083

Values are presented as mean ± standard deviation; *p*-values are determined by *t*-test or chi-squared test.

**Table 2:** Association between *TMEM232* (rs17132261) and infant atopic dermatitis.

Genotype	aOR	95% CI	*p*-Value
TT	Ref		
TC	2.77	1.39–5.51	0.004
CC	3.45	1.75–6.79	<0.001

Adjusted for maternal age and feeding method.

**Table 3:** Infant and maternal biochemical and environmental factors based on *TMEM232* genotype.

	CC		TC		TT			
	N	Mean ± SD or %	N	Mean ± SD or %	N	Mean ± SD or %	*p*-Value ^1^	*p*-Value ^2^
Infant								
WBC	568	9.12(±2.57)	411	8.96(±2.7)	79	8.85(±2.67)	0.518	0.748
Monocytes, %	568	8.1(±2.95)	407	7.91(±2.93)	79	7.66(±3.27)	0.362	0.112
Lymphocytes, %	568	61.76(±11.48)	407	63.49(±9.81)	79	63.17(±10.53)	0.041	0.080
Neutrophils, %	568	26.38(±10.77)	405	24.95(±9.69)	79	25.43(±9.87)	0.100	0.336
Eosinophils, %	568	3.08(±2.17)	407	3.05(±2.41)	79	3.11(±1.94)	0.970	0.562
Basophils, %	568	0.53(±0.39)	406	0.52(±0.34)	79	0.5(±0.51)	0.826	0.726
Total IgE	576	64.02(±167.71)	395	66.18(±160.03)	80	47.33(±68.02)	0.625	0.067
Egg IgE	576	1.46(±6.67)	395	1.85(±7.64)	80	1.21(±3.02)	0.603	0.273
Feeding method	617	92.1	449	91.1	90	94.7	0.470	0.564
Mother								
Age	686	33(±3.51)	497	33.36(±3.6)	94	33.54(±3.81)	0.129	0.252
WBC	565	8.94(±2.72)	416	9.05(±2.85)	84	9.4(±3.85)	0.375	0.182
Monocytes, %	437	6.82(±1.9)	332	6.7(±2.03)	65	6.82(±1.93)	0.661	0.154
Lymphocytes, %	437	21.05(±8.79)	334	20.71(±7.82)	65	19.24(±6.08)	0.253	0.506
Neutrophils, %	432	70.68(±10.02)	327	71(±9.23)	65	72.74(±7.03)	0.265	0.527
Eosinophils, %	436	1.13(±1.09)	329	1.23(±1.91)	65	0.94(±0.58)	0.293	0.525
Basophils, %	434	0.21(±0.17)	329	0.22(±0.17)	64	0.19(±0.13)	0.526	0.746
Total IgE	548	107.02(±320.48)	404	103.14(±278.17)	74	147.51(±254.86)	0.499	0.395
Der f IgE	439	5.07(±12.74)	332	4.98(±12.73)	65	4.9(±13.77)	0.993	0.049
SPT Der f	248	40.3	174	39.2	40	45.5	0.549	0.258
History of allergic disease	217	31.6	166	33.3	32	33.7	0.788	0.252

^1^, *p*-values are determined by ANOVA; ^2^, *p*-values are determined using regression adjusted for maternal age and feeding method.

**Table 4:** Offspring’s clinical data by TMEM232 genotype and maternal factor.

	CC + TC				TT					
	+		−		+		−			
	N	Mean ± SD	N	Mean ± SD	N	Mean ± SD	N	Mean ± SD	*p*-Value ^1^	*p*-Value ^2^
History of allegic disease										
WBC	312	9.15(±2.7)	667	9.01(±2.58)	30	8.66(±2.83)	49	8.97(±2.59)	0.72	0.874
Monocytes, %	312	8.04(±3.1)	663	8.01(±2.87)	30	7.7(±3.37)	49	7.64(±3.25)	0.78	0.244
Lymphocytes, %	312	62.7(±10.34)	663	62.38(±11.08)	30	63.81(±10.66)	49	62.78(±10.55)	0.885	0.551
Neutrophils, %	312	25.3(±9.33)	661	26.01(±10.8)	30	25(±10.28)	49	25.69(±9.71)	0.757	0.818
Eosinophils, %	312	3.18(±2.16)	663	3.02(±2.32)	30	3.04(±1.98)	49	3.15(±1.93)	0.769	0.537
Basophils, %	312	0.55(±0.38)	662	0.51(±0.36)	30	0.45(±0.32)	49	0.53(±0.6)	0.33	0.486
Total IgE	313	76.81(±202.67)	658	59.23(±142.72)	32	52.34(±67.71)	48	43.99(±68.73)	0.317	0.046
Egg IgE	313	2(±9.12)	658	1.44(±5.87)	32	1.38(±3.51)	48	1.09(±2.67)	0.63	0.348
SPT-Der f										
WBC	354	9.02(±2.8)	556	9.08(±2.51)	33	9.48(±2.98)	43	8.33(±2.37)	0.244	0.456
Monocytes, %	350	8.28(±3.11)	556	7.94(±2.87)	33	8.22(±4.08)	43	7.26(±2.59)	0.119	0.040
Lymphocytes, %	350	62.05(±11.33)	556	62.73(±10.56)	33	62.12(±10.16)	43	64.16(±10.15)	0.591	0.552
Neutrophils, %	349	25.8(±10.44)	555	25.72(±10.31)	33	26.08(±8.9)	43	24.67(±9.56)	0.917	0.973
Eosinophils, %	350	3.15(±2.15)	556	3.03(±2.37)	33	3.13(±2.07)	43	3.12(±1.9)	0.875	0.755
Basophils, %	350	0.54(±0.41)	555	0.52(±0.35)	33	0.45(±0.34)	43	0.55(±0.62)	0.492	0.653
Total IgE	349	72.41(±188.69)	538	61.37(±149.12)	33	46.28(±63.21)	43	48.5(±74.66)	0.593	0.177
Egg IgE	349	1.81(±6.79)	538	1.36(±6.97)	33	1.06(±2.87)	43	1.29(±3.26)	0.757	0.434

+, the presence of history of allergic disease or SPT-Der f; −, the absence of history of allergic disease or SPT-Der f; ^1^, *p*-values are determined by ANOVA; ^2^, *p*-values are determined using linear regression adjusted for maternal age, and feeding method.

**Table 5:** Association between *TMEM232* (rs17132261) and infant atopic dermatitis based on maternal factors.

Genotype	aOR	95% CI	*p*-Value
No history of allergic disease			
TT	Ref		
TC	2.09	0.95–4.57	0.067
CC	2.70	1.25–5.83	0.012
History of allergic disease			
TT	Ref		
TC	5.78	1.33–25.22	0.031
CC	6.69	1.55–28.89	0.020
No sensitization to Der f			
TT	Ref		
TC	2.12	0.71–6.33	0.178
CC	3.72	1.28–10.85	0.016
Sensitization to Der f			
TT	Ref		
TC	4.85	1.09–21.57	0.038
CC	4.91	1.12–21.63	0.035

Adjusted for maternal age and feeding method.

## Data Availability

The datasets generated and/or analyzed during the current study are available from the corresponding author upon reasonable request. The data are not publicly available due them containing information that could compromise research participant privacy. The SNP information are available at this links: https://www.ncbi.nlm.nih.gov/snp/?term=rs17132261, https://www.ebi.ac.uk/gwas/search?query=rs17132261 accessed on 15 September 2024.

## Supplementary Materials

The following supporting information can be downloaded at: https://www.mdpi.com/article/10.3390/genes15111446/s1, Table S1. Association between *TMEM232* SNPs and infant atopic dermatitis. Table S2. Comparison of maternal factors according to infant’s atopic dermatitis. Table S3. Characteristics of maternal clinical data with maternal factors.

## Abbreviations

AD	Atopic dermatitis
TMEM232	Transmembrane protein 232
GWAS	Genome-wide association studies
COCOA	The Cohort for Childhood Origin of Asthma and Allergic Disease
ISAAC	International Study of Asthma and Allergies in Childhood
SPT	Skin prick test
SCORAD	SCORing Atopic Dermatitis
Der f	Dermatophagoides farinae
SD	Standard deviation
OR	Odds ratios
aOR	Adjusted odds ratios
TMEM	Transmembrane proteins
