# Supplementary material for: Effects of TMEM232 Variant on Infant Atopic Dermatitis According to Maternal Factors
Source: Genes (Basel). 2024 Nov 8;15(11):1446. doi: 10.3390/genes15111446 (PMC11593446; doi:10.3390/genes15111446)
Supplement: Supplementary file 1 [file genes-15-01446-s001.zip › genes-3262952-supplementary.pdf]

Supplementary Table S1. Association between TMEM232 SNPs and infant atopic dermatitis

CHR	SNP	Position	A1	A2	p-value	OR
5	rs36806	109272682	T	C	0.0266	1.25
5	-	109280664	G	A	0.0424	1.32
5	rs149995409	109330056	T	G	0.0206	1.84
5	rs78660913	109714737	C	T	0.0248	1.41
5	rs2081909	109724995	A	G	0.0248	0.78
5	rs72788430	109800443	A	G	0.0056	1.77
5	rs146786747	109878130	G	A	0.0480	0.66
5	rs28516873	109921239	G	A	0.0236	1.32
5	rs17132261	110008214	T	C	0.0004	0.69
5	rs72773143	110011369	C	A	0.0215	1.70

Supplementary Table S2. Comparison of maternal factors according to infant's atopic dermatitis

	Non-AD		AD		
	N	%	N	%	p-value
Mother					
History of allergic disease	427	30.5	173	34.9	0.083
SPT Der f	478	38.2	178	43.0	0.097
SPT Cat	111	8.9	48	11.7	0.112
SPT Dog	120	9.6	52	12.6	0.105
Pet	85	6.3	25	5.2	0.424
Drinking status	106	8.0	26	5.7	0.113
Passive smoking	371	27.7	131	27.2	0.884
Delivery mode	873	62.6	317	64	0.601

p-values are determined by chi-squared test.

Supplementary Table S3. Characteristics of maternal clinical data with maternal factors

	-		+		p-value
	N	Mean ± SD or %	N	Mean ± SD or %	
History of allergic disease					
WBC	1,018	9.07(±2.66)	472	9.17(±2.79)	0.509
Monocytes, %	1,012	7.86(±2.73)	472	8.03(±3.2)	0.3
Lymphocytes, %	1,012	62.6(±10.86)	472	62.58(±10.68)	0.971
Neutrophils, %	1,009	25.9(±10.56)	472	25.62(±9.87)	0.628
Eosinophils, %	1,012	3.03(±2.21)	471	3.08(±2.02)	0.681
Basophils, %	1,011	0.52(±0.37)	469	0.54(±0.37)	0.231
Total IgE	1,008	62.85(±177.39)	463	73(±180.51)	0.311
Egg IgE	1,008	1.28(±5.19)	463	1.87(±8.21)	0.097
Spt Der f					
WBC	839	9.03(±2.58)	537	9.18(±2.83)	0.318
Monocytes, %	837	7.8(±2.71)	533	8.12(±3.08)	0.045
Lymphocytes, %	837	63.02(±10.38)	533	61.97(±11.27)	0.078
Neutrophils, %	835	25.55(±10.11)	532	26.2(±10.47)	0.25
Eosinophils, %	837	3.01(±2.16)	532	3.06(±2.05)	0.711
Basophils, %	834	0.52(±0.37)	532	0.53(±0.39)	0.714
Total IgE	815	66.49(±188.34)	524	67.37(±173.56)	0.931
Egg IgE	815	1.3(±6.26)	524	1.59(±6.11)	0.397

Values are presented as mean ± standard deviation.
p-values are determined by t-test.
